# Correlated 5-Hydroxymethylcytosine (5hmC) and Gene Expression Profiles Underpin Gene and Organ-Specific Epigenetic Regulation in Adult Mouse Brain and Liver

**DOI:** 10.1371/journal.pone.0170779

**Published:** 2017-01-26

**Authors:** I-Hsuan Lin, Yi-Fan Chen, Ming-Ta Hsu

**Affiliations:** 1 VYM Genome Research Center, National Yang-Ming University, Taipei, Taiwan; 2 The Center of Translational Medicine, Taipei Medical University, Taipei, Taiwan; 3 The Ph.D. Program for Translational Medicine, College of Medical Science and Technology, Taipei Medical University, Taipei, Taiwan; 4 Institute of Biomedical Informatics, National Yang-Ming University, Taipei, Taiwan; 5 Chien-Tien Hsu Cancer Research Foundation, Taipei, Taiwan; National Center for Toxicological Research, UNITED STATES

## Abstract

**Background:**

DNA methylation is an epigenetic mechanism essential for gene regulation and vital for mammalian development. 5-hydroxymethylcytosine (5hmC) is the first oxidative product of the TET-mediated 5-methylcytosine (5mC) demethylation pathway. Aside from being a key intermediate in cytosine demethylation, 5hmC may have potential regulatory functions with emerging importance in mammalian biology.

**Methods:**

Here, we investigate the global 5hmC enrichment in five brain structures, including cerebellum, cerebral cortex, hippocampus, hypothalamus and thalamus, as well as liver tissues from female and male adult mice by using chemical capture-based technique coupled with next-generation sequencing. At the same time, we carried out total RNA sequencing (RNA-seq) to analyze the transcriptomes of brain regions and liver tissues.

**Results:**

Our results reveal preferential 5hmC enrichment in the gene bodies of expressed genes, and 5hmC levels of many protein-coding genes are positively correlated with RNA expression intensity. However, more than 75% of genes with low or no 5hmC enrichment are genes encode for mitochondrial proteins and ribosomal proteins despite being actively transcribed, implying different transcriptional regulation mechanisms of these housekeeping genes. Brain regions developed from the same embryonic structures have more similar 5hmC profiles. Also, the genic 5hmC enrichment pattern is highly tissue-specific, and 5hmC marks genes involving in tissue-specific biological processes. Sex chromosomes are mostly depleted of 5hmC, and the X inactive specific transcript (*Xist*) gene located on the X chromosome is the only gene to show sex-specific 5hmC enrichment.

**Conclusions:**

This is the first report of the whole-genome 5hmC methylome of five major brain structures and liver tissues in mice of both sexes. This study offers a comprehensive resource for future work of mammalian cytosine methylation dynamics. Our findings offer additional evidence that suggests 5hmC is an active epigenetic mark stably maintained after the global reprogramming event during early embryonic development.

## Introduction

Mammalian transcriptional regulation is complex and involves multiple layers of coordinated control mechanisms to ensure proper function at cellular and organismal levels. Epigenetic modifications of DNA and histone proteins are regarded as key players in epigenetic regulation of gene expression. In some cases, DNA methylation of *cis*-regulatory elements can hinder recruitment of transcription factors. [1,2] The formation of transcriptionally silent heterochromatin involved DNA methylation and repressive histone modifications such as H3K27me3 and H3K9me3. [3,4] Other than transcriptional regulation, DNA methylation plays important roles in diverse biological processes, such as embryonic development, genomic imprinting, X-chromosome inactivation, differentiation and cancer development.

DNA methylation is a process whereby a methyl (CH_3_) group is covalently attached to the C5 position of a cytosine residue by DNA methyltransferases. The 5-methylcytosine (5mC) is the major form of DNA modification. Since the discovery of 5mC, multiple oxidized 5mC variants have been identified. These variants are the products of sequentially oxidation of 5mC to 5-hydroxymethylcytosine (5hmC), 5hmC to 5-formylcytosine (5fC), and 5fC to 5-carboxylcytosine (5caC) by TET family of cytosine oxygenases. Subsequently, thymine-DNA glycosylase can excise 5fC and 5caC and replace with unmodified cytosines by base excision repair (BER) pathway. [5,6,7] The DNA demethylation process plays an essential role in early embryogenesis. After fertilization, both maternal and paternal genomes are reprogrammed to totipotency. [8,9,10,11,12] TET3-dependent active demethylation and passive DNA replication-dependent dilution of 5mC and its oxidized derivatives are major contributors of genome-wide erasure of DNA methylation in mouse zygotes. Global DNA demethylation also happens during establishment of the primordial germ cells which will develop into sperm or oocyte. [13,14]

5hmC is the most widely studied DNA methylation variants. Not only is 5hmC an essential intermediate of active DNA demethylation process in early developmental stages, studies have also shown evidence of 5hmC being a stable epigenetic modification after epigenetic reprogramming and is detectable in different tissues and cell types in various levels. [15,16,17,18] In mouse, central nervous system and spinal cord have relatively higher levels of 5hmC, followed by kidney, nasal epithelium, bladder, heart, skeletal muscle and lung, whereas liver, spleen, testes and pituitary gland have lowest 5hmC levels. [19] In human, 5hmC levels is high in brain, liver, kidney and colorectal tissues, lower in lung and lowest in heart, breast and placenta. [20] Recently, Lunnon et al assayed the 5hmC in a total of 26 human prefrontal cortex and cerebellum samples using Illumina 450K methylation array. They found the human cerebellum has higher 5hmC than the prefrontal cortex and regions that exhibited greater differences between the two brain regions are enriched at CpG islands and in the gene bodies. [21] The region- and tissue-specific accumulation of 5hmC in terminally differentiated cells suggests its involvement in the epigenetic regulation of mammalian systems. Indeed, studies have shown accumulation of 5hmC modifications at active euchromatic regions, *cis*-regulatory elements and intragenic regions of genes with intermediate or high levels of expression. [22,23,24,25,26] Moreover, several proteins can recognize and bind to 5hmC to affect the expression levels of 5hmC-marked genes. Examples of 5hmC reader includes UHRF1 (ubiquitin-like with PHD and ring finger domains), MBD3 (methyl-CpG binding domain protein 3), MeCP2 (Methyl-CpG-binding protein 2), RPL26 (ribosomal protein L26), PRPF8 (pre-mRNA processing factor 8), MSH6 (MutS homolog 6) and THAP11 (THAP domain containing 11). [24,27,28,29]

There is growing evidence that 5hmC is a distinct epigenetic mark and interacts with chromatin binding proteins to play a functional role in transcriptional regulation. 5hmC also plays a role in cancer biology and was implicated in the regulation in tumorigenesis. The global reduction of 5hmC levels and mutation of TET enzymes were frequently observed in cancers. [30,31,32] The change in 5hmC pattern has been shown to regulate gene expression in cancer cells, and this characteristic has given 5hmC the potential to be considered as an important diagnostic and prognostic marker for cancer. [33,34,35,36,37]

In this study, we profiled and analyzed the whole-genome 5hmC enrichment and gene expression patterns of five brain structures (i.e. cerebellum, cerebral cortex, hippocampus, hypothalamus and thalamus) and liver tissues of female and male adult mice to determine the biological role of 5hmC methylation. The standard sodium bisulfite-based sequencing does not distinguish between 5mC and 5hmC. [38] Therefore, we used the chemical capture-based technique to enrich 5hmC methylated DNA fragments followed by next-generation sequencing to investigate the relationship between 5hmC enrichment and gene expression between different brain structures, organs as well as sexes. Our data show that 5hmC accumulates in the intragenic regions of actively transcribed genes and is depleted in sex chromosomes. 5hmC enrichment pattern is highly organ-specific that marks genes with brain- or liver-specific expression. Furthermore, we show that there are exceptions to this rule especially genes of housekeeping functions. 5hmC methylation is an important constituent of the DNA demethylation pathways that contributes to epigenetic plasticity, and may play a key role in early central nervous system development and maintaining proper cellular transcriptional activity into adulthood.

## Results

### Profiling of 5hmC enriched regions

**Fig 1:**
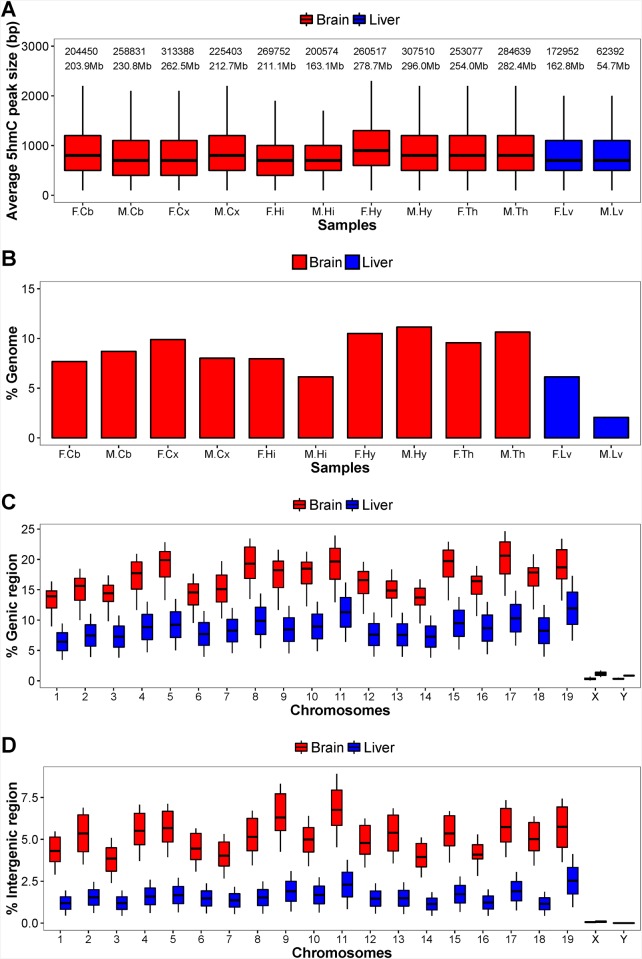
5hmC enrichment statistics in mouse brain and liver tissues. (A) The distribution of the size of 5hmC enriched regions in the 12 samples. (B) The percentage genome that is 5hmC-enriched. The per-chromosome 5hmC enrichment of the (C) intragenic regions and (D) intergenic regions in brain and liver samples. The sample names are abbreviated. F and M denote female and male samples respectively. Cb = cerebellum; Cx = cortex; Hi = hippocampus; Hy = hypothalamus; Th = thalamus; Lv = liver.

We used a capture-based method to obtained 5hmC-enriched DNA fragments from cerebellum, cerebral cortex, hippocampus, hypothalamus and thalamus as well as liver tissues of female and male adult mice. Next-generation sequencing (NGS) and peak calling of the enriched regions against input controls were performed on the twelve samples and the basic stats were presented in S1 Table. The average size of a 5hmC enriched region is about 800 bp (Fig 1A). The sum of enriched region is about 240 Mb in brain, which covers approximately 9% of the genome. Liver samples generally have fewer 5hmC enriched regions than brain samples, especially in male liver where 5hmC-enriched regions cover only 2% of the genome (Fig 1B). The 5hmC enriched regions preferentially located in the intragenic and promoter regions. The proportions of 5hmC in gene body and intergenic regions vary between chromosomes. Fig 1C showed the percent coverage in intragenic regions of autosomes range from 9% to 25% (average 17%) in brain and 3% to 17% in liver (average 9%). The 5hmC-enrichment are much lower in intergenic regions compared to intragenic regions in both organs, where the average percentage converge in brain and liver are 5% and 2% respectively (Fig 1D). The sex chromosomes are mostly without 5hmC enrichment.

**Fig 2:**
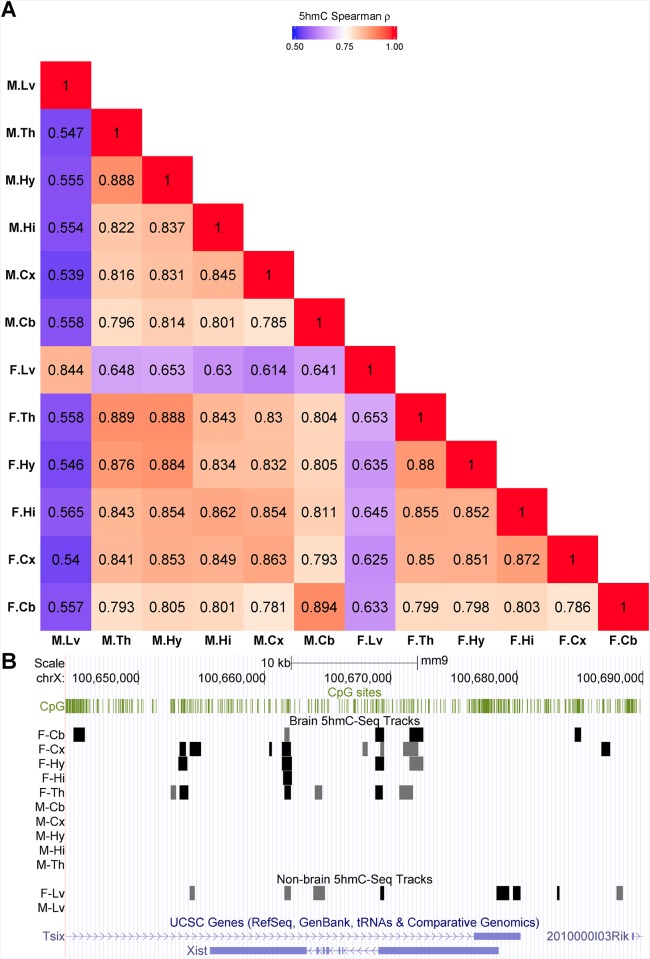
5hmC enrichment profile similarity among the 12 mouse samples. (A) An unsorted similarity matrix of intragenic 5hmC coverage. The colors represent the correlation scores (i.e., similarity) ranging from dark blue (ρ = 0.5) to dark red (ρ = 1.0). (B) Visualization of the genomic region around *Xist*. The 5hmC-enriched regions for each sample are shown in separate labeled track. The sample names are abbreviated. F and M denote female and male samples respectively. Cb = cerebellum; Cx = cortex; Hi = hippocampus; Hy = hypothalamus; Th = thalamus; Lv = liver.

We use Spearman rank correlation as a measure of pair-wise similarity of intragenic 5hmC enrichment between samples. Fig 2A showed the brain and liver samples is moderately correlated (ρ 0.55 ~ 0.65), whereas higher correlation is observed among samples from the same organ (ρ > 0.78). The matrix also showed cerebellum samples have highly correlated enrichment patterns (ρ = 0.89), and the four hypothalamus and thalamus samples are also highly correlated (ρ > 0.97). The Spearman's rho values reflect the degree of similarity between different brain regions and that between brain and liver, in term of epigenetic mechanisms as well as the biological and developmental implications.

The 5hmC enrichment patterns among samples of the same sex are less correlated than that between the same region or organ. We searched for genes that showed sex-specific enrichment pattern, and identified the X inactive specific transcript (*Xist*) to be the only gene to display sex-specific differential 5hmC pattern in both brain and liver samples with 5hmC modification only in female samples (see Fig 2B).

### 5hmC enrichment is found in specific gene biotypes and molecule types

**Fig 3:**
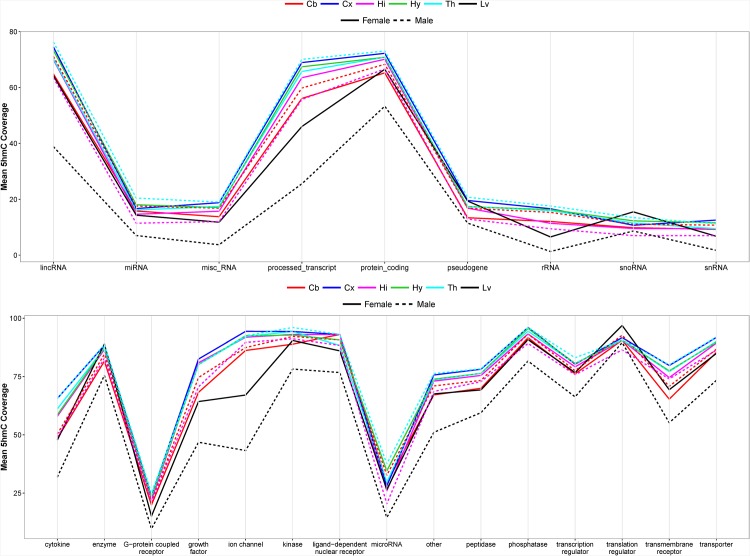
Average intragenic 5hmC coverage of genes classified by (A) gene biotype and (B) molecule type in 12 mouse brain and liver samples. The categorical data points were joined and shown as line graphs to allow visualization of the 5hmC enrichment trends across biotypes and molecule types among the 12 samples. The sample names are abbreviated. Cb = cerebellum; Cx = cortex; Hi = hippocampus; Hy = hypothalamus; Th = thalamus; Lv = liver.

We use the gene biotype annotation provided by GENCODE to determine if 5hmC enrichment is common in all biotypes. The sex chromosomes contain very few 5hmC enrichment as shown in Fig 1C and 1D, hence genes on chrX and chrY were excluded from the following analyzes. 5hmC enrichment is found in an average of 68.5% of the intragenic regions of protein coding genes, processed transcripts and long intergenic non-coding RNAs (lincRNAs) in brain samples, and 49.0% in liver samples (Fig 3A). On the other hand, less than 20% of intragenic regions of pseudogenes and short non-coding RNAs in both organs are 5hmC-enriched. We also classify genes according to the ingenuity pathway analysis (IPA) molecule type annotation and calculate the average intragenic 5hmC coverage in each type. Most molecule types have more than 50% average intragenic 5hmC coverage, except G-protein coupled receptors and microRNAs which have lowest intragenic 5hmC enrichment (Fig 3B).

**Fig 4:**
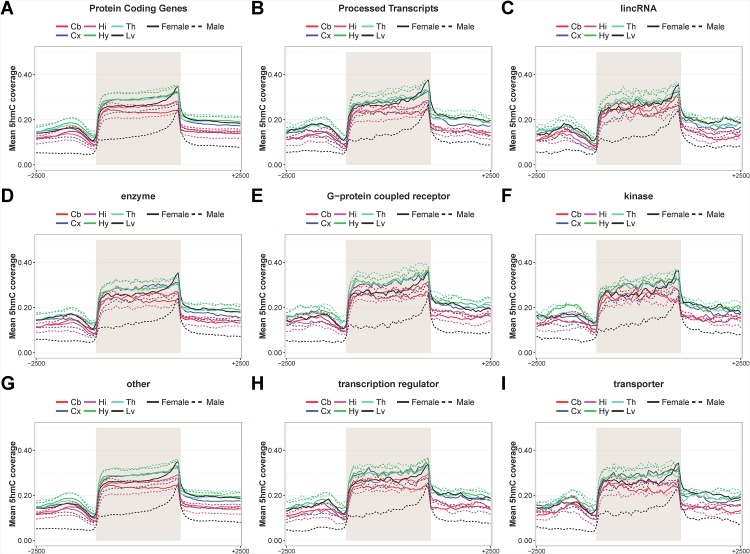
5hmC enrichment in relation to intragenic regions across 12 samples. We evaluate the intragenic 5hmC enrichment patterns in (A) protein-coding genes, (B) process transcripts, (C) lincRNAs, (D) enzymes, (E) G-protein coupled receptors, (F) kinases (G), others, (H) transcription regulators and (I) transporters. The 5hmC coverage within gene bodies and up to 2.5 kb upstream of transcription start site (TSS) and downstream of transcription termination site (TTS) are shown. Gray box represents the gene body from TSS to TTS. The sample names are abbreviated. Cb = cerebellum; Cx = cortex; Hi = hippocampus; Hy = hypothalamus; Th = thalamus; Lv = liver.

We analyze the position of 5hmC enrichment within and around gene body to determine its preferred location. The intragenic 5hmC enrichment patterns are similar between different gene groups (Fig 4A to 4I). The average 5hmC coverage along gene body is stable between some of the brain regions than between sexes. For example, the patterns between female hypothalamus and thalamus, as well as between male hypothalamus and thalamus, are highly similar. The overall patterns fluctuate most between organs, and the male liver samples have much lower intragenic 5hmC than other samples. The average 5hmC enrichment is highest towards transcription termination site (TTS; 40%) and lowest at transcription start site (TSS; 10%) in all of the investigated biotypes and molecule types.

### Intragenic 5hmC enrichment correlates with gene expression levels

**Fig 5:**
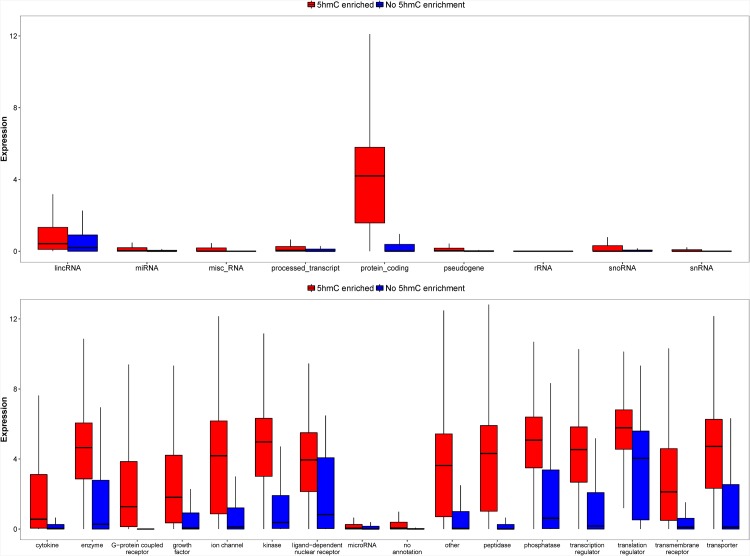
Box plots representation of RNA expression levels of genes classified by (A) gene biotypes and (B) molecule types in the 12 mouse brain and liver samples. The box plots show medians and interquartile ranges of the expression intensities. The red and blue colors represent genes with and without intragenic 5hmC enrichment respectively. Statistical differences between genes with and without 5hmC in the nine biotypes are indicated with asterisks (* *P* < 0.05; ** *P* < 1e-5; *** *P* < 1e-10; Wilcoxon rank sum test). All molecule types showed significance statistical differences (*P* < 1e-5; Wilcoxon rank sum test).

We have shown that the 5hmC enrichment varies substantially across gene biotypes, molecule types and organs. Therefore, we examine if the amount of intragenic 5hmC enrichment associates with gene expression levels. We performed total RNA sequencing (RNA-seq) on two biological replicates of the five brain regions and liver tissue in adult female and male mice. The gene expression level is represented as log2 read count-per-million (log2 CPM). Protein coding genes and lincRNAs that have intragenic 5hmC enrichment are significantly more highly expressed than that without 5hmC enrichment (*P* < 2.2e-16; Fig 5A). Other gene biotypes are lowly expressed regardless of 5hmC enrichment status. All the molecule types showed higher RNA expression in genes containing intragenic 5hmC enrichment than those without (Fig 5B). The microRNA and genes without annotation were more lowly expressed than other molecule types.

**Fig 6:**
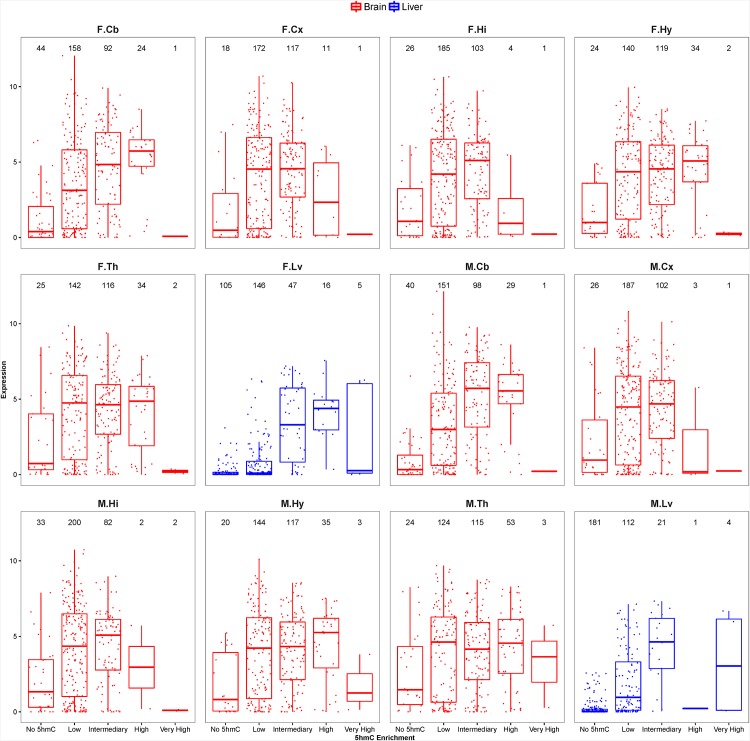
Box plots representation of intragenic 5hmC enrichment versus RNA expression levels of 319 ion channel genes. The distribution expression intensities of genes with no (0%), low (<20%), intermediary (20 ~ 50%), high (50% ~ 80%), and very high (> 80%) intragenic 5hmC enrichment. The number of genes in each 5hmC enrichment class is labeled above the corresponding box plot. The brain and liver sample are color in red and blue colors respectively. The sample names are abbreviated. F and M denote female and male samples respectively. Cb = cerebellum; Cx = cortex; Hi = hippocampus; Hy = hypothalamus; Th = thalamus; Lv = liver.

In Fig 3B we showed that the ion channel genes have lower intragenic 5hmC coverage in liver samples compared with brain samples. Therefore, we examine the gene expression levels of ion channels in the two organs to determine if liver samples express fewer ion channel mRNAs. Fig 6 showed liver samples had more genes with no and low intragenic 5hmC enrichment, and these were significantly more lowly expressed (*P* < 0.05). In brain and liver samples, genes that have no or low intragenic 5hmC coverage are also more lowly expressed (Fig 6 and S1 Fig). Of the 319 genes encoding the ion channel proteins, between 188 to 219 genes were moderately to highly expressed (log2 CPM ≥ 2.5) in brain samples. There are only 60 and 65 genes that have log2 CPM value of 2.5 or more in liver samples. Hence, more than 80% of the ion channel genes were either lowly or not expressed in liver and the intragenic 5hmC enrichment is congruently low in these genes.

### Intragenic 5hmC enrichment correlates with organ-specific expression

**Fig 7:**
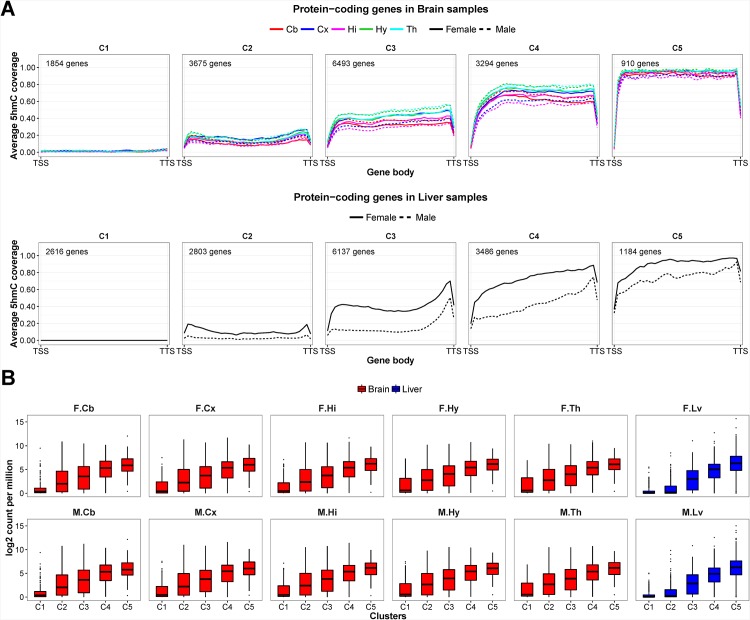
Intragenic 5hmC enrichment patterns and expression intensities of five clusters of protein-coding genes. (A) The protein-coding genes are classified into five clusters according to 5hmC enrichment patterns within gene body (from TSS to TTS). The clusters are arranged with increase 5hmC coverage from C1 to C5. The number of genes in each cluster is given in each subplot. (B) The box plots representation of RNA expression levels of genes in each cluster in the 12 mouse brain and liver samples. The sample names are abbreviated. F and M denote female and male samples respectively. Cb = cerebellum; Cx = cortex; Hi = hippocampus; Hy = hypothalamus; Th = thalamus; Lv = liver.

Protein coding genes that have high intragenic 5hmC enrichment also have high RNA expression levels. We then determine if intragenic 5hmC enrichment pattern is associated with gene expression by performing hierarchical clustering to group protein-coding genes with similar 5hmC patterns into clusters. Fig 7A and 7B showed five clusters of genes that have gradual increase in the intragenic 5hmC enrichment, and more intragenic 5hmC corresponds with increasing gene expression.

We performed gene ontology (GO) enrichment analysis on the genes in the five clusters and assessed the biological processes enriched in each of the cluster using topGO. Genes that have low intragenic 5hmC and low expression in brain (i.e. Brain Cluster 1; C1) have GO terms such as regulation of transcription, regulation of RNA biosynthetic process, chromatin assembly and regulation of gene expression. Brain C2 and C3 comprise genes that are moderately 5hmC-enriched and expressed. They are associated with metabolic-related processes. Genes from the Brain C4 are associated with cellular component organization, localization, protein modification process and intracellular signal transduction. Genes that have highest intragenic 5hmC and highly expressed play major roles in nervous system development, generation of neurons, neurogenesis and developmental process.

On the contrary, liver have different enriched biological processes in each cluster compared to brain. The least 5hmC-enriched and expressed genes are associated with GO terms such as cell-cell signaling, neuropeptide signaling pathway, nervous system development, generation of neurons and neuron differentiation. Liver C2 contains genes mapped to cell projection organization, nervous system development, synaptic transmission and ion transmembrane transport. The most significant processes assigned to Liver C3, C4 and C5 clusters are mainly metabolic-related processes, such as cellular metabolic process, organic substance metabolic process and primary metabolic process. The disparities in the distribution of biological process in various clusters in brain and liver samples indicate 5hmC enrichment is part of the epigenetic information that plays a role in organ-specific functions and maintenances.

To complement the GO enrichment analysis, we used ingenuity pathway analysis (IPA) to look for functions associated with C1 and C5 clusters of brain and liver samples. Genes in Brain C5 mapped predominantly to brain-specific functions, such as development of neurons, neuritogenesis, morphogenesis of neurites and development of central nervous system. Top functions associated with least 5hmC-enriched and expressed Brain C1 genes include activation of DNA endogenous promoter, quantity of cells and transcription. Similar to findings from GO analysis, genes in Liver C5 have important roles in lipid metabolism, such as concentration of lipid, morphology of liver and transport of lipid as the top 15 functions of Liver C5. Whereas, neurological functions such as behavior, morphology of nervous system, sensation and neurotransmission are associated with Liver C1 genes. The results of functional annotations from IPA and GO enrichment analysis are consistent. It indicates special biological consequences of 5hmC methylation in multicellular organisms.

### 5hmC enrichment patterns of housekeeping genes are not correlated with gene expression

Hierarchical clustering using 5hmC enrichment pattern of protein-coding genes shows that it positively correlates with the level of gene expression. To determine if this is a general phenomenon, we select genes that are annotated as transcription regulators, enzymes (including kinases, peptidases and phosphatases) and receptors and transporters (including transporters, ion channels, ligand-dependent nuclear receptors, transmembrane receptors, and G-protein coupled receptors) and perform hierarchical clustering accordingly. We showed in S2 to S4 Figs that the genes from these three subsets are more highly expressed as intragenic 5hmC increases from one cluster to the next in all samples. Hence, the correlation between 5hmC and gene expression is common in the major molecule types in both brain and liver samples.

**Fig 8:**
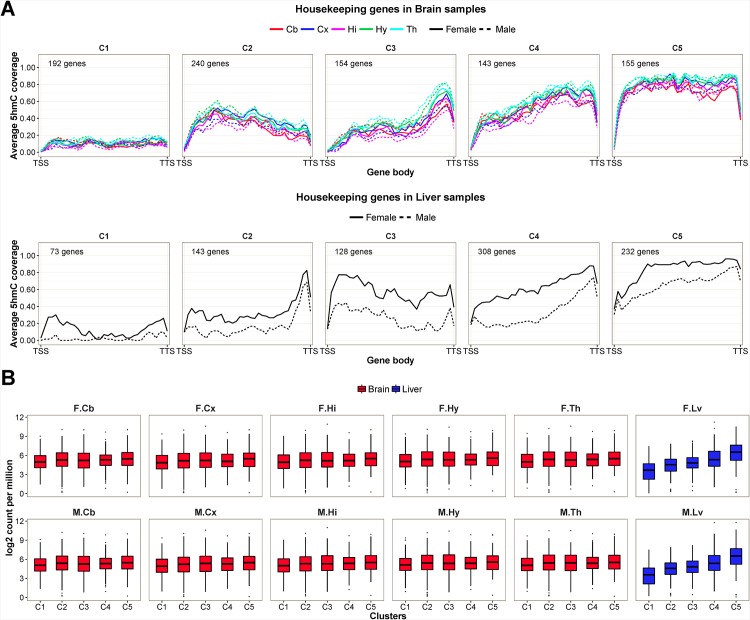
Intragenic 5hmC enrichment patterns and expression intensities of five clusters of housekeeping genes. (A) The housekeeping genes are classified into five clusters according to 5hmC enrichment patterns within gene body (from TSS to TTS). The clusters are arranged with increase 5hmC coverage from C1 to C5. The number of genes in each cluster is given in each subplot. (B) The box plots representation of RNA expression levels of genes in each cluster in the 12 mouse brain and liver samples. The sample names are abbreviated. F and M denote female and male samples respectively. Cb = cerebellum; Cx = cortex; Hi = hippocampus; Hy = hypothalamus; Th = thalamus; Lv = liver.

In multicellular organisms, housekeeping genes encode proteins that are produced constantly in cells from most if not all of the organs. These proteins are required for the proper maintenance of basic functionality of cells. We collected 1439 human housekeeping proteins curated by The Human Protein Atlas database, [39] identified their homologs in mouse and study if the genes encoding for the homologs have high amount of intragenic 5hmC enrichment in our datasets. A total of 925 mouse homologs from the five well-known classes of housekeeping proteome (i.e. citric acid cycle related proteins, RNA polymerase related proteins, ribosomal proteins, cytoskeleton related proteins and mitochondrial proteins) are used to perform clustering. Surprisingly, housekeeping genes are all highly expressed but there is a large variation of intragenic 5hmC levels (Fig 8A and 8B). In both brain and liver samples, the C1 and C5 clusters represent subset of housekeeping genes that have the least and most amount of intragenic 5hmC respectively. Yet, the distribution of expression intensities between C1 and C5 remain similar in brain samples. Smaller changes are observed among housekeeping genes in liver samples compared to the three gene subsets (S1 to S3 Figs). This shows 5hmC methylation may be less involved in the transcriptional regulation of housekeeping genes, particularly genes in C1 of the brain samples (S2 Table).

We examine the distribution of the classes of housekeeping genes in the five 5hmC clusters. Of the five classes of housekeeping genes, the genes encoding for mitochondrial proteins and ribosomal proteins has dissimilar cluster assignment in brain and liver. Of the 540 genes encoding for mitochondrial proteins, 294 genes are assigned to C1 and C2 clusters in brain, whereas 134 genes are assigned to C1 and C2 clusters in liver. This means that, despite being highly expressed, 54% of the genes encoding for mitochondrial proteins are mostly depleted of or low with 5hmC enrichment in brain, whereas only 25% are lowly 5hmC-enriched in liver. This phenomenon is also observed in ribosomal proteins, where 78 of 126 genes (62%) are assigned to C1 and C2 clusters in brain and only 29 genes (23%) are assigned to C1 and C2 clusters in liver. The mitochondrial and ribosomal genes accounts for 372 genes (86%) in brain C1 and C2, and 163 genes (75%) in liver C1 and C2.

### Exceptions of the positive correlation between 5hmC enrichment and gene expression

We observed the expression of housekeeping genes are not well correlated with intragenic 5hmC enrichment. To identify genes besides housekeeping genes whose expression does not correlate with 5hmC enrichment, we systematically analyze our 5hmC and RNA-seq datasets and identified two classes of exceptions of protein-coding genes: (1) 326 genes that are expressed but without 5hmC enrichment and (2) 192 genes that are not expressed but with 5hmC enrichment in brain and liver samples (S3 Table). We performed functional analysis using IPA. Based on computed P-values, the top 2 significant categories associated to the first class of genes are "Cellular Assembly and Organization" and "DNA Replication, Recombination, and Repair". Of the 326 genes without intragenic 5hmC enrichment but are expressed, 18 genes (*Cd207*, *Dock2*, *Dpt*, *Hgf*, *Hist1h1c*, *Hist1h1d*, *Hist1h1e*, *Junb*, *Mad2l1*, *Mcm4*, *Mif*, *Mt1*, *Rhob*, *Saa1*, *Socs6*, *Thbs2*, *Tlr4*, and *Tmsb10*) are mapped to the "Cellular Assembly and Organization" category and 12 genes (*Fancg*, *H2afx*, *Hist1h1c*, *Hist1h1d*, *Hist1h1e*, *Lig4*, *Mad2l1*, *Mybl1*, *Poll*, *Prdm9*, *Socs6*, and *Tlr4*) are mapped to "DNA Replication, Recombination, and Repair" category.

The two categories associated with the second class of genes are "Molecular Transport" and "Cellular Development". Genes mapped to "Molecular Transport" are *Aqp3*, *Calhm1*, *Camp*, *Cyp8b1*, *Epo*, *Gpbar1*, *Il13*, *Mpo*, *Rsc1a1*, and *Slc34a1*, whereas genes mapped to the "cellular Development" are *Camp*, *Cebpe*, *Edn2*, *Epo*, *Gfi1b*, *Gpr3*, *Il13*, *Il17c*, *Il9r*, *Mmel1*, *Nr2e3*, and *Prph2*. The P-values computed by IPA are higher in these cases than with the previous assessments, such as the significant biological functions associated with genes obtained from hierarchical clustering analyses. It implies less significance in the function enrichment of these two classes of genes. Also 52 (16%) and 83 (43%) genes in first and second class respectively are novel genes without canonical names. Hence, the true significance of these exceptions remained to be investigated.

### Functional annotation of genes with organ-specific gene expression

**Fig 9:**
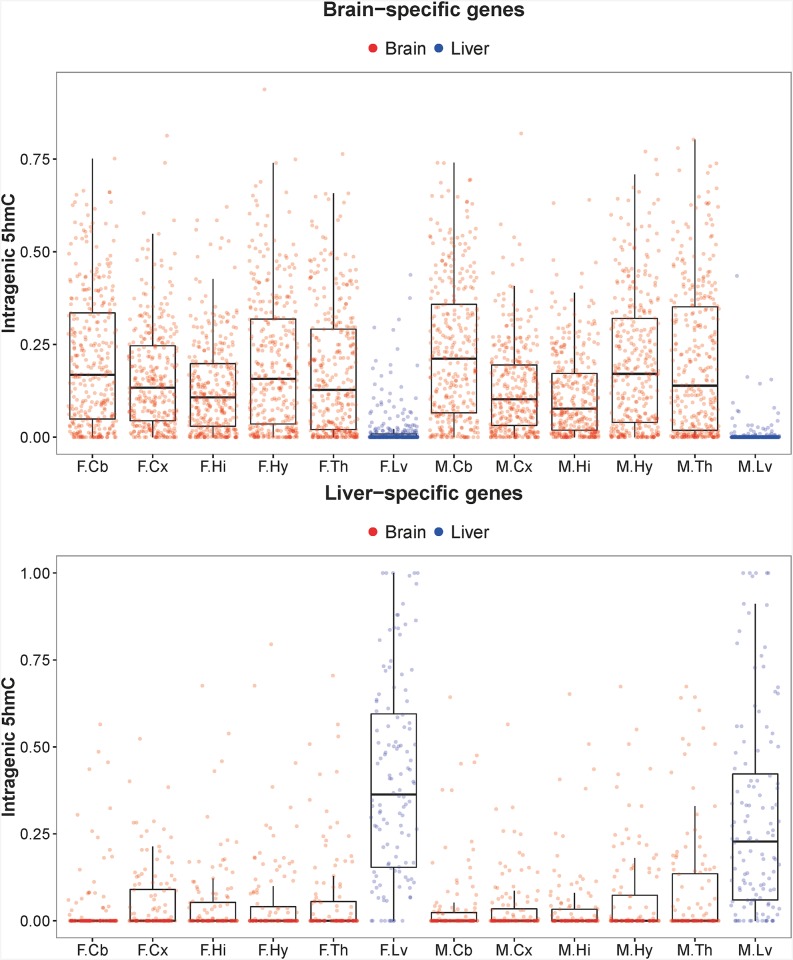
Tissue-specific gene expression concomitant with intragenic 5hmC enrichment. Box plots showing the distribution of proportion of gene bodies that overlap 5hmC-enriched peaks in genes expressed only in (A) brain and (B) liver samples. Each point represents a tissue-specific gene, and there are 331 brain-specific genes shown in (A) and 124 liver-specific genes shown in (B). The sample names are abbreviated. F and M denote female and male samples respectively. Cb = cerebellum; Cx = cortex; Hi = hippocampus; Hy = hypothalamus; Th = thalamus; Lv = liver. There were significant differences in 5hmC enrichment levels between the two tissue groups assessed by one-way ANOVA in both tissue-specific gene sets (*P* < 0.05).

We identify organ-specific genes based on RNA-seq data from the five brain regions and liver samples. We plot the distribution of intragenic 5hmC coverage of brain-specific and liver-specific genes (Fig 9A and 9B). We showed that genes with brain-specific expression have significant higher 5hmC enrichment in brain samples and likewise liver-specific genes have significant higher 5hmC enrichment in liver samples (one-way ANOVA, *P* < 0.05). This result fosters the notion that there is a strong positive relationship between 5hmC enrichment and gene expression, especially in genes that are required for organ/tissue-specific activities.

GO enrichment analysis of the 331 brain-specific and 124 liver-specific genes identify specialized functions and localization relating to each organ-specific gene sets. Brain-specific genes partake in synaptic function and neurotransmission. The brain-specific genes were most enriched with protein localized at neuron, synapse, synaptic membrane, cell projection and axon. These proteins participate in processes such as synaptic transmission, cell-cell signaling, transmembrane transport, nervous system development and neuron development (S4 Table).

In contrast, the liver-specific genes encode protein with high metabolic activities. The top biological process categories include monocarboxylic acid metabolic process, carboxylic acid metabolic process, organic acid metabolic process, small molecule metabolic process and oxidation-reduction process. Liver proteins are predominantly enriched in extracellular space and regions, endoplasmic reticulum and endoplasmic reticulum membrane (S5 Table).

### Expression status of ten-eleven translocation (Tet) genes and known readers for 5hmC

**Fig 10:**
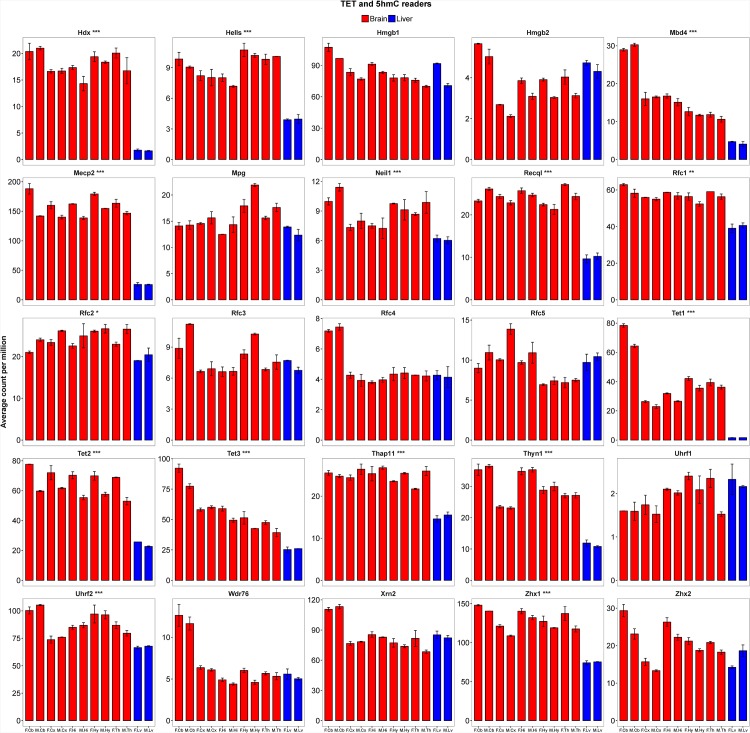
The expression intensities of Tet genes and 5hmC readers. Bar plots showing the average RNA expression levels of *Tet1*, *Tet2*, *Tet3* and 22 5hmC readers represented as read count-per-million. Error bars show the 95% confidence intervals of the means. Statistical differences between brain and liver samples are indicated with asterisks (* *P* < 1e-2; ** *P* < 1e-3; *** *P* < 1e-5; Two Sample t-test). The sample names are abbreviated. F and M denote female and male samples respectively. Cb = cerebellum; Cx = cortex; Hi = hippocampus; Hy = hypothalamus; Th = thalamus; Lv = liver.

Our RNA-seq data showed the *Tet1* is highly expressed in all five brain parts, especially in cerebellum samples, but not in liver tissue (Fig 10). *Tet2* and *Tet3* are expressed in all twelve samples; however, their expressions are slightly lower in liver samples compared to brain samples. We also evaluate the expression status of 22 genes previously identified as 5hmC readers. [29] Eleven of the 22 readers, including *Hdx*, *Hells*, *Mbd4*, *Mecp2*, *Neil1*, *Recql*, *Rfc1*, *Rfc2*, *Thap11*, *Thyn1*, and *Zhx1* are significantly over-expressed in brain compared to liver samples (*P* < 1e-3). Among these, the X-linked methyl-CpG-binding protein 2 (Mecp2) has been shown to activate gene expression by increasing neural chromatin accessibility. [24] As the major 5hmC-binding protein in the brain, mutation in *Mecp2* causes Rett syndrome. Many of 5hmC readers are identified in neuronal progenitor cells and adult mouse brain [29] and our data show that half of the readers exhibit brain-specific over-expression. However little is known of their functional roles in the central nervous system (CNS).

## Discussion

DNA methylation is one of the key epigenetic modification involving in transcriptional regulation. The 5mC and 5hmC are the best-studied epigenetic marks of the four DNA methylation variants (i.e. 5mC, 5hmC, 5fC and 5caC). In this work, we use next-generation sequencing technology to identify genomic regions that are enriched with 5hmC in five mouse brain structures (i.e. cerebellum, cerebral cortex, hippocampus, hypothalamus and thalamus) and liver tissues. 5hmC is more enriched at intragenic regions in both brain and liver samples. 5hmC is higher in all five brain structures compared to liver. This is consistent with previous findings that showed adult brain has the highest amount of 5hmC of all the organs and tissues in human and mouse. [15,19,20,40] The five brain structures of both female and male mice have an average of 9% of genome that is enriched with 5hmC, whereas only 6% and 2% of the genome were 5hmC-enriched in female and male liver respectively.

Although 5hmC is depleted in sex chromosomes, the *Xist* gene located on chrX is enriched with 5hmC in female but not in male samples. The Xist protein is the master regulator of X inactivation where it coats the entire inactive chromosome to induce gene silencing. [41] *Xist* is expressed only on the inactive chromosome in female mammals. Our data showed only the female *Xist* intragenic regions to contain 5hmC enrichment, implying only the inactive chromosome to contain 5hmC methylation. Other than *Xist*, we did not find other genes showing sex-specific 5hmC enrichment.

We compare the 5hmC patterns between different brain structures. As a whole, the genome-wide 5hmC enrichment patterns are fairly conserved among various brain structures. Using correlation as a similarity measure, we can group the five brain structures into three larger parts, i.e. (1) thalamus and hypothalamus, (2) cortex and hippocampus, and (3) cerebellum. The 5hmC patterns of cerebellums are less similar than the rest of the four structures. This distinction coincides with the fact that cerebellum is derived from the rhombencephalon (hindbrain), whereas the other parts are from prosencephalon (forebrain) during the three-vesicle stage of embryonic brain development. As development proceeds, the prosencephalon is divided into two sections. The telencephalon eventually developed into structures that include cerebral cortex and hippocampus, and diencephalon is composed of thalamus and hypothalamus. The 5hmC in cells from different adult brain structures retain the characteristics of major divisions of brain during prenatal stages. This suggests 5hmC deposition is a highly coordinated process during brain development. This result has an interesting implication in the regulation of brain development or functions by 5hmC.

The accumulation of 5hmC in human and mouse brain begins early in prenatal period. Yamaguchi et al showed that 5hmC is detectable in E9.5 mouse embryo and E11.5 mouse primordial germ cells. [42] Hahn et al found 5hmC is higher in maturing neurons than neural progenitor cells and young neurons during neuronal differentiation (between E11.5 to E15.5) in mouse. [43] The accumulation of 5hmC persists from fetus, after birth into adulthood in mammalian brain. [44,45,46] The agreement between degree of similarity of 5hmC pattern among mouse brain structures and brain vesicles as early as E9 to E9.5 not only suggests the 5hmC is one of the epigenetic modifications important in brain development, it also implies the presence of a mechanism capable of regulating the accumulation in an organ- and cell-specific fashion, and maintaining genomic 5hmC into adulthood.

Mecp2 is highly abundant in brain. It binds to 5hmC to facilitate gene expression and mutation of this gene can result in neurodevelopmental disorders. [24,47,48] A report by Spruijt et al identified additional 5hmC binding proteins in mouse embryonic stem cells, neuronal progenitor cells and adult mouse brain. [29] The recruitment of Mecp2 to 5hmC-enriched genes is evidently part of the epigenetic regulation of neural chromatin structure and gene activity in brain. Other than *Mecp2*, our RNA-seq data revealed eleven 5hmC readers over-expressed in the five brain structures. The roles of these readers in CNS remained to be elucidated.

5hmC accumulates over time and it appears to occur preferentially in the gene body of actively transcribed genes. The presence of 5hmC in gene body is a hallmark associated with active genes in both brain and liver samples. Using ion channels as example, we showed more than 80% of 319 genes are lowly or not expressed in liver and have low or no intragenic 5hmC enrichment. On the other hand, brain expresses many of these ion channels and 5hmC are concordantly high in these genes. Furthermore, we illustrate the positive correlation between 5hmC enrichment and RNA abundance in various molecule types of protein-coding genes. We interrogate four genes sets (i.e protein-coding genes, transcription regulators, enzymes and membrane proteins) and group genes with similar intragenic enrichment pattern using hierarchical clustering and assess gene expression levels of each cluster. Not surprisingly, genes in cluster that has the lowest intragenic 5hmC enrichment are least expressed, while genes in cluster with highest 5hmC throughout the gene body are highly expressed. This observation is in agreement with earlier reports in different human and mouse tissues whereby intragenic 5hmC levels positively correlate with gene expression. [43,44,49,50]

In the four gene sets, the average 5hmC is always higher towards the 3' end of the gene in the intermediary cluster. Could this reflect a preference of 5hmC deposition to begin at the end of a gene and continues towards the transcription start site remain to be determined. It is an effective indicator of organ/tissue-specific genes because 5hmC marks active genes. Genes that are expressed in either brain or liver samples are enriched with intragenic 5hmC in the respective organ. Functional evaluation with GO terms and IPA showed these genes participate in functions that are characteristics of the given organ. Thus the dynamic accumulation and modulation of intragenic 5hmC are important epigenetic processes in assisting transcriptional networks in multicellular organisms. [51,52,53,54] Interestingly, intragenic 5hmC is not correlated with gene expression levels of housekeeping genes. Our assessment of 925 mouse homologs of human housekeeping genes showed they have similar high expression profiles regardless of varying degrees of intragenic 5hmC enrichment. Specifically, there are twice as many genes encode for mitochondrial and ribosomal proteins in brain than in liver to show no or low 5hmC enrichment even though the genes are actively expressed. Thus, gene expression is regulated by other means despite 5hmC is not enriched in several housekeeping genes. As cells accumulate more 5hmC over its lifespan, it is critical for cells to have multiple robust regulatory mechanisms to maintain its proper cellular functions.

We used capture-based sequencing to show 5hmC accumulates in the gene body of actively transcribed genes and marks organ-specific genes in adult mouse brain and liver. Its relevance in organ-specificity and gene expression suggests 5hmC is not merely an intermediate in the DNA demethylation process but an essential component of the epigenome of animals throughout the life course. Our findings strengthen the link between 5hmC enrichment and gene expression for most protein-coding genes but also identify the housekeeping genes to be the exceptions of this association.

## Materials and Methods

### Mice

All animal work was carried out in strict accordance with the appropriate guidelines approved by the Institutional Animal Care and Use Committee (IACUC) at Taipei Medical University (Permit Number: LAC-2014-0141). Wild-type male and female mice were purchased from National Laboratory Animal Center (NLAC, Taiwan). All animals were maintained under specific-pathogen-free conditions with a 12-h light, 12-h dark cycle, and provided regular chow diet (LabDiet 5010) and water *ad libitum*. Mice of both sexes at 3 months of age were used in the experiments. Mice were euthanized by carbon dioxide inhalation. All efforts were made to minimize suffering. Five brain sub-regions (cortex, hippocampus, thalamus, hypothalamus, and cerebellum) and liver were harvested, frozen in liquid nitrogen and stored at −80°C before use.

### Genomic DNA extraction

Mouse tissues acquired from two biological replicates were digested in NTES buffer [10 mM Tris-HCl (pH 8.0), 1 mM EDTA, 1 M NaCl, and 0.5% SDS] and 200 μg/ml proteinase K overnight at 55°C. The mixture was extracted twice with phenol-chloroform. The genomic DNA was precipitated with ethanol, and dissolved in autoclaved water.

### RNA extraction

Mouse tissues acquired from two biological replicates were homogenized in TRIzol reagent (Thermo Fisher Scientific). The mixture was extracted twice with phenol-chloroform. Total RNA was precipitated with isopropanol and treated with DNase I to remove contaminating genomic DNA. The reaction was stopped by addition of EDTA and incubation at 70°C for 5 min. Subsequently, total RNA was precipitated with ethanol twice to remove chemicals and dissolved in autoclaved water.

### Enrichment of 5hmC DNA fragments

Purified genomic DNA was fragmented to 100–500 bp using Bioruptor (Diagenode). An aliquot of sheared genomic DNA was saved as input control for sequencing to determine the baseline peaks. 5hmC enrichment was perform on the sheared DNA using the Hydorxymethyl Collector kit (Active Motif) according to manufacturer's instruction. Briefly, one microgram of fragmented DNA was incubated in the presence of a beta-glucosyltransferase and a modified UDP-glucose donor to add a glucosyl-moiety to 5hmC. The modified glucosyl-5hmC was then conjugated with biotin and captured by magnetic streptavidin beads followed by eluting the enriched DNA from the biotin linker. The resulting enriched 5hmC DNA was then subjected to sequencing library construction.

### Library construction and sequencing of 5hmC-enriched and input DNA

10 ng of enriched 5hmC DNA and sheared genomic DNA without 5hmC enrichment were end-repaired, A-tailed, and ligated to adaptors using the TruSeq ChIP Sample Preparation kit (Illumina). The adapter-ligated 5hmC library was amplified by PCR for 10 cycles using KAPA HiFi DNA polymerase (Kapa Biosystems). The enriched 5hmC library was sequenced on HiSeq2500 (Illumina) by paired-end sequencing with 100 bp read length.

### Library construction and sequencing of total RNA

Ribosomal RNA was depleted from 1 mg of total RNA with RIN (RNA integrity number) ranged from 8.0–10 using Ribo-Zero Gold Kit (Human/Mouse/Rat) (Epicentre). The sequencing library for rRNA-depleted RNA was prepared using TruSeq Stranded mRNA Sample Preparation Kit (Illumina) which produced directional RNA-seq libraries. The adapter-ligated double-stranded cDNA library was enriched with 6 to 7 cycles of PCR using KAPA HiFi DNA polymerase. The stranded RNA-seq library was sequenced on HiSeq2500 by single end sequencing with 100 bp read length.

### 5hmC-seq data processing

The 100-bp paired-end raw reads were checked for quality using FastQC (v0.11.5). [55] Reads were mapped to the mouse reference genome (mm9) with the BWA-MEM (v0.7.15) algorithm. [56] Duplicates were removed with Picard MarkDuplicates (v 1.124) and name-sorted with Picard SortSam. [57] Uniquely-mapped BAM alignments were converted to BED format with bedtools bamToBed (v2.25.0). [58] The 5hmC-enriched regions (peaks) were identified using SICER (v1.1) [59] with the following parameters: redundancy threshold = 1, window size = 100 bp, fragment size = 200 bp, gap size = 100 & 200 bp, and FDR = 0.01. GENCODE annotation (vM1) was used to annotate 5hmC peaks and downstream analyses. [60] The 5hmC-seq data have been deposited in the ArrayExpress database under accession number E-MTAB-5167.

### RNA-seq data processing

The 100-bp single end raw reads were checked for quality using FastQC (v0.11.5). Reads were mapped to the mouse genome (GENCODE vM1) using STAR (v2.4.0c). [61] The featureCounts program of the Subread package (v1.4.6) was used to summarize reads counts over gene features.[62] The RNA-seq data have been deposited in the ArrayExpress database under accession number E-MTAB-5166.

### Bioinformatics and statistical analyses

Comparison and arithmetic operations between features and mouse genome were performed with bedtools. Statistical analysis and graphics were performed in the R environment. Pairwise Spearman rank correlation of 5hmC enrichment pattern between samples was computed with cor(). R Bioconductor package topGO (v2.22.0) was used to perform Gene Ontology enrichment analysis with given gene sets. [63] R Bioconductor package edgeR (v3.12.1) was used to normalize read count data and determine differential gene expression between tissues. [64]

## Supporting Information

S1 Fig Statistical analysis of intragenic 5hmC enrichment patterns and expression intensities of ion channel genes. Differences between intragenic 5hmC enrichment and gene expression of ion channel genes in brain and liver samples were analyzed by two-way ANOVA, followed by post-hoc analysis with Tukey's honest significant difference (HSD) test to determine which group means differ significantly. (A) The normal probability plot of residuals. (B) The pair-wise comparisons with Tukey's HSD showing confidence intervals for the difference in the means for all ten pairs of groups. Groups that had confidence intervals not containing 0 were considered to have significant different true mean gene expression levels, and were colored in red.
(TIF)

S2 Fig Intragenic 5hmC enrichment patterns and expression intensities of five clusters of transcription regulators. (A) The genes encode for transcription regulators are classified into five clusters according to 5hmC enrichment patterns within gene body (from TSS to TTS). The clusters are arranged with increase 5hmC coverage from C1 to C5. The number of genes in each cluster is given in each subplot. (B) The box plots representation of RNA expression levels of genes in each cluster in the 12 mouse brain and liver samples. The sample names are abbreviated. F and M denote female and male samples respectively. Cb = cerebellum; Cx = cortex; Hi = hippocampus; Hy = hypothalamus; Th = thalamus; Lv = liver.
(TIF)

S3 Fig Intragenic 5hmC enrichment patterns and expression intensities of five clusters of enzymes. (A) The genes encode for kinases, peptidases and phosphatases are classified into five clusters according to 5hmC enrichment patterns within gene body (from TSS to TTS). The clusters are arranged with increase 5hmC coverage from C1 to C5. The number of genes in each cluster is given in each subplot. (B) The box plots representation of RNA expression levels of genes in each cluster in the 12 mouse brain and liver samples. The sample names are abbreviated. F and M denote female and male samples respectively. Cb = cerebellum; Cx = cortex; Hi = hippocampus; Hy = hypothalamus; Th = thalamus; Lv = liver.
(TIF)

S4 Fig Intragenic 5hmC enrichment patterns and expression intensities of five clusters of receptors and transporters. (A) The genes encode for transporters, ion channels, ligand-dependent nuclear receptors, transmembrane receptors, and G-protein coupled receptors are classified into five clusters according to 5hmC enrichment patterns within gene body (from TSS to TTS). The clusters are arranged with increase 5hmC coverage from C1 to C5. The number of genes in each cluster is given in each subplot. (B) The box plots representation of RNA expression levels of genes in each cluster in the 12 mouse brain and liver samples. The sample names are abbreviated. F and M denote female and male samples respectively. Cb = cerebellum; Cx = cortex; Hi = hippocampus; Hy = hypothalamus; Th = thalamus; Lv = liver.
(TIF)

S1 Table 5hmC sequencing and mapping statistics of twelve mouse samples. The sample names are abbreviated. F and M denote female and male samples respectively. Cb = cerebellum; Cx = cortex; Hi = hippocampus; Hy = hypothalamus; Th = thalamus; Lv = liver.
(XLSX)

S2 Table 192 housekeeping genes that are expressed in brain samples but with no or very low intragenic 5hmC enrichment. These genes are assigned to C1 cluster after performing hierarchical clustering using 5hmC enrichment pattern of 925 mouse homologs of human housekeeping proteins curated by The Human Protein Atlas database.
(XLSX)

S3 Table List of genes that showed gene expression but without 5hmC enrichment or not expressed but with 5hmC enrichment in the brain and liver samples. (XLSX)

S4 Table GO term enrichment analyses of brain-specific genes with topGO. (XLSX)

S5 Table GO term enrichment analyses of liver-specific genes with topGO. (XLSX)
