# Supplementary figures and images for: Correlated 5-Hydroxymethylcytosine (5hmC) and Gene Expression Profiles Underpin Gene and Organ-Specific Epigenetic Regulation in Adult Mouse Brain and Liver
Source: PLoS One. 2017 Jan 26;12(1):e0170779. doi: 10.1371/journal.pone.0170779 (PMC5268415; doi:10.1371/journal.pone.0170779)

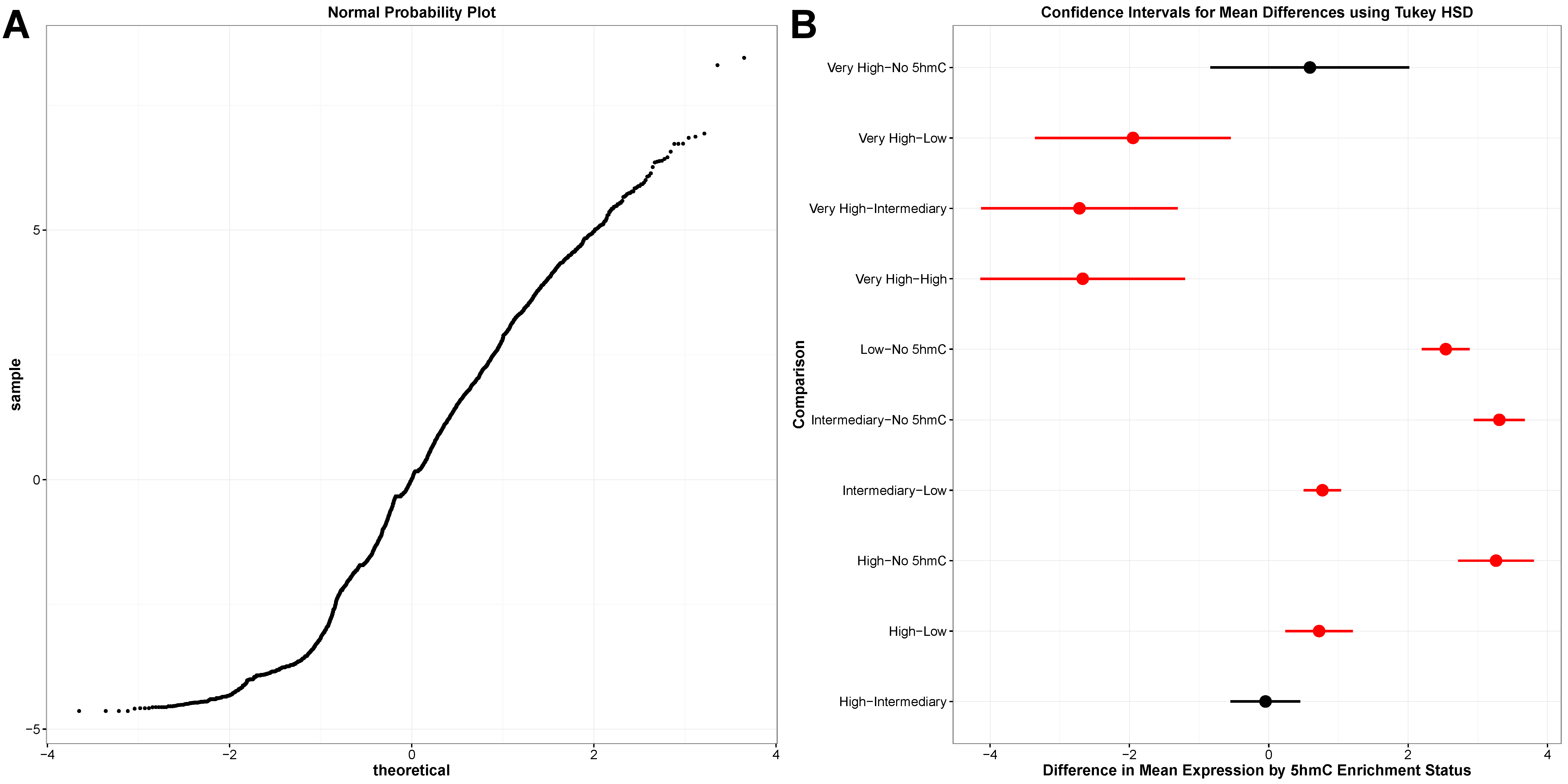

Supplement: S1 Fig — Differences between intragenic 5hmC enrichment and gene expression of ion channel genes in brain and liver samples were analyzed by two-way ANOVA, followed by post-hoc analysis with Tukey's honest significant difference (HSD) test to determine which group means differ significantly. (A) The normal probability plot of residuals. (B) The pair-wise comparisons with Tukey's HSD showing confidence intervals for the difference in the means for all ten pairs of groups. Groups that had confidence intervals not containing 0 were considered to have significant different true mean gene expression levels, and were colored in red. (TIF) [file pone.0170779.s001.tif]

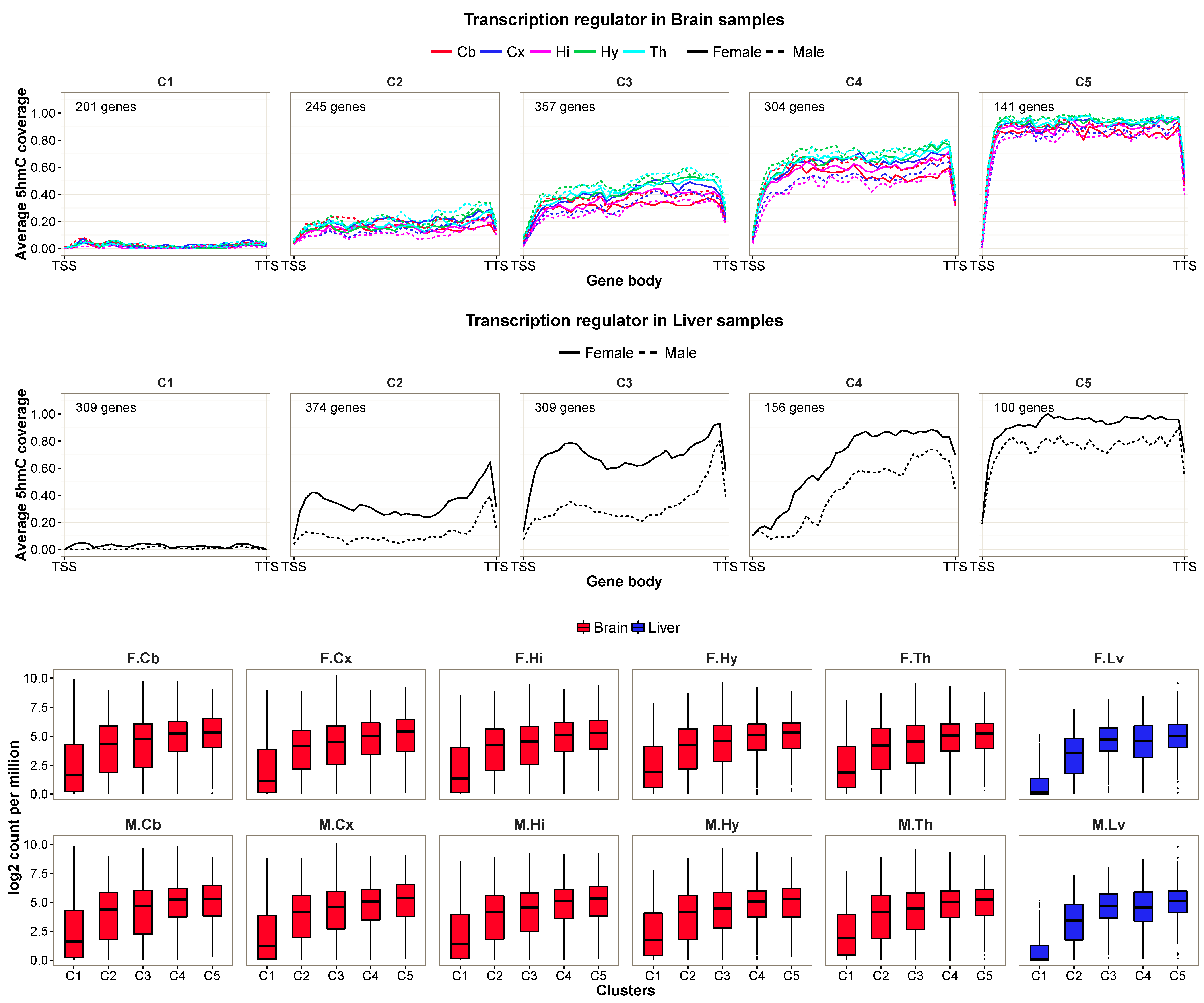

Supplement: S2 Fig — (A) The genes encode for transcription regulators are classified into five clusters according to 5hmC enrichment patterns within gene body (from TSS to TTS). The clusters are arranged with increase 5hmC coverage from C1 to C5. The number of genes in each cluster is given in each subplot. (B) The box plots representation of RNA expression levels of genes in each cluster in the 12 mouse brain and liver samples. The sample names are abbreviated. F and M denote female and male samples respectively. Cb = cerebellum; Cx = cortex; Hi = hippocampus; Hy = hypothalamus; Th = thalamus; Lv = liver. (TIF) [file pone.0170779.s002.tif]

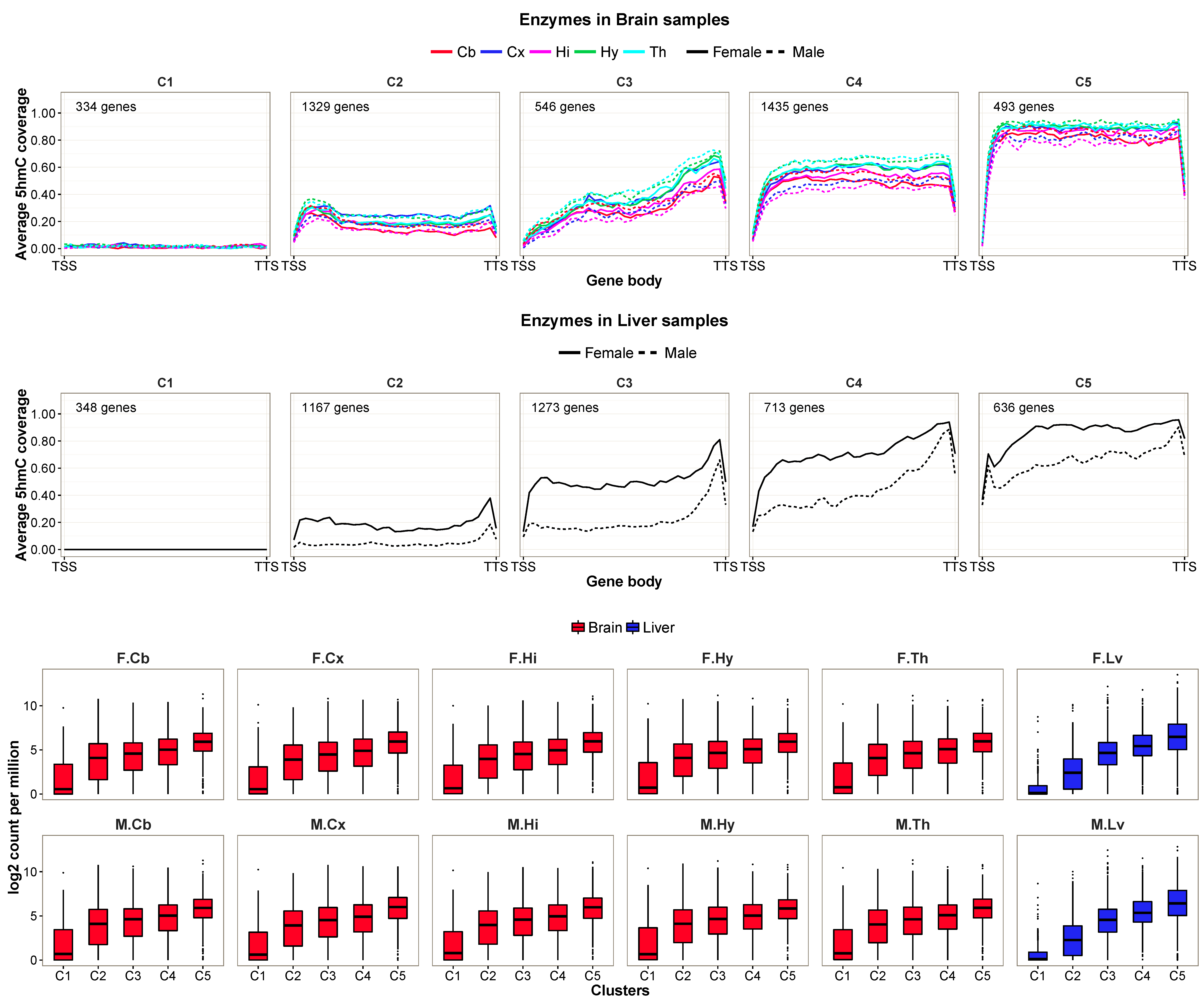

Supplement: S3 Fig — (A) The genes encode for kinases, peptidases and phosphatases are classified into five clusters according to 5hmC enrichment patterns within gene body (from TSS to TTS). The clusters are arranged with increase 5hmC coverage from C1 to C5. The number of genes in each cluster is given in each subplot. (B) The box plots representation of RNA expression levels of genes in each cluster in the 12 mouse brain and liver samples. The sample names are abbreviated. F and M denote female and male samples respectively. Cb = cerebellum; Cx = cortex; Hi = hippocampus; Hy = hypothalamus; Th = thalamus; Lv = liver. (TIF) [file pone.0170779.s003.tif]

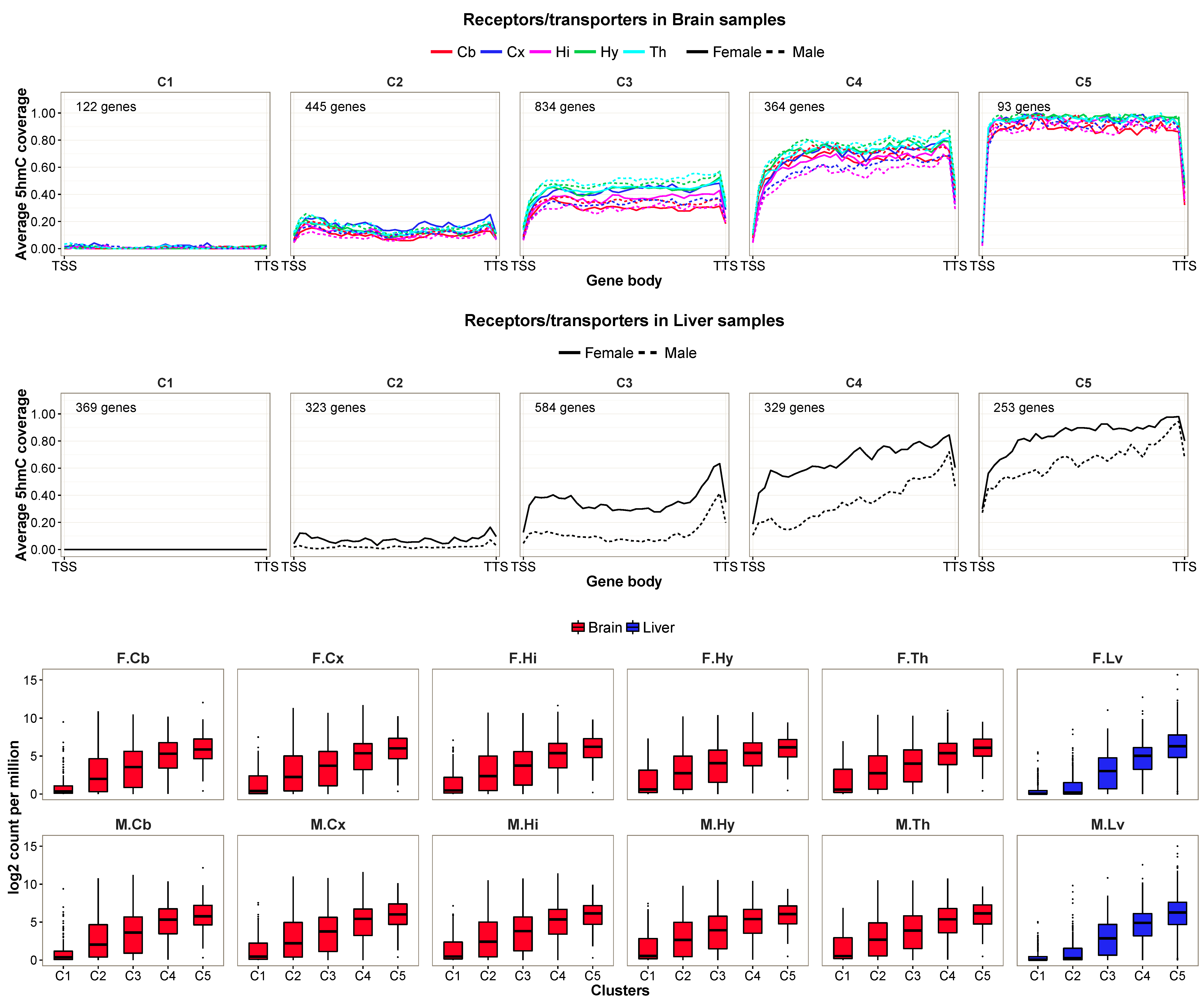

Supplement: S4 Fig — (A) The genes encode for transporters, ion channels, ligand-dependent nuclear receptors, transmembrane receptors, and G-protein coupled receptors are classified into five clusters according to 5hmC enrichment patterns within gene body (from TSS to TTS). The clusters are arranged with increase 5hmC coverage from C1 to C5. The number of genes in each cluster is given in each subplot. (B) The box plots representation of RNA expression levels of genes in each cluster in the 12 mouse brain and liver samples. The sample names are abbreviated. F and M denote female and male samples respectively. Cb = cerebellum; Cx = cortex; Hi = hippocampus; Hy = hypothalamus; Th = thalamus; Lv = liver. (TIF) [file pone.0170779.s004.tif]
